# Ascaroside Pheromones: Chemical Biology and Pleiotropic Neuronal Functions

**DOI:** 10.3390/ijms20163898

**Published:** 2019-08-09

**Authors:** Jun Young Park, Hyoe-Jin Joo, Saeram Park, Young-Ki Paik

**Affiliations:** 1 Interdisciplinary Program in Integrative Omics for Biomedical Science, Yonsei University, Seoul 03722, Korea; 2 Yonsei Proteome Research Center, Yonsei University, Seoul 03722, Korea; 3 Department of Chemical Physiology and Dorris Neuroscience Center, The Scripps Research Institute, La Jolla, CA 92037, USA

**Keywords:** ascaroside pheromone, *C. elegans*, dauer, neuronal signaling, sexual behavior, survival signals, stress response

## Abstract

Pheromones are neuronal signals that stimulate conspecific individuals to react to environmental stressors or stimuli. Research on the ascaroside (ascr) pheromones in *Caenorhabditis elegans* and other nematodes has made great progress since ascr#1 was first isolated and biochemically defined in 2005. In this review, we highlight the current research on the structural diversity, biosynthesis, and pleiotropic neuronal functions of ascr pheromones and their implications in animal physiology. Experimental evidence suggests that ascr biosynthesis starts with conjugation of ascarylose to very long-chain fatty acids that are then processed via peroxisomal β-oxidation to yield diverse ascr pheromones. We also discuss the concentration and stage-dependent pleiotropic neuronal functions of ascr pheromones. These functions include dauer induction, lifespan extension, repulsion, aggregation, mating, foraging and detoxification, among others. These roles are carried out in coordination with three G protein-coupled receptors that function as putative pheromone receptors: SRBC-64/66, SRG-36/37, and DAF-37/38. Pheromone sensing is transmitted in sensory neurons via DAF-16-regulated glutamatergic neurotransmitters. Neuronal peroxisomal fatty acid β-oxidation has important cell-autonomous functions in the regulation of neuroendocrine signaling, including neuroprotection. In the future, translation of our knowledge of nematode ascr pheromones to higher animals might be beneficial, as ascr#1 has some anti-inflammatory effects in mice. To this end, we propose the establishment of ***pheromics*** (*pher*omone *omics*) as a new subset of integrated disciplinary research area within chemical ecology for system-wide investigation of animal pheromones.

## 1. What Are Pheromones?

Pheromones are neuronal signaling molecules synthesized by various organisms and then excreted into the environment, where they typically stimulate individuals of the same species to react to environmental changes (e.g., temperature shifts, biological stimuli, or nutritional changes) [1,2]. It is thought that most organisms, from prokaryotes to higher animals such as humans, can produce and use pheromones for communication between conspecific individuals. In most cases, pheromones trigger neuronal events that are linked to various behavioral responses. The outcomes of such neuronal stimulation are the modulation of developmental and/or physiological programs that can support adaptation to new environments [3]. For example, approximately 1500 insect pheromones have been identified since bombykol was discovered in 1959 [4]. These pheromones mediate common behaviors such as courtship rituals, mating, aggregation, dispersal (e.g., spacing or epideictic pheromones), alarm, recruitment (e.g., trailing pheromones), and maturation [2,4]. In mammals, pheromones are used for marking territories, and for signaling mating and feeding preparedness [5,6].

In humans, there have been numerous reports of putative pheromones; however, their existence has not been experimentally confirmed. For example, a putative human pheromone was proposed to be excreted from the apocrine gland in the male underarm, although its functions have not been characterized [7,8]. Unlike other mammals, humans lack a functional vomeronasal organ (VNO), which processes pheromonal signals in mice and other vertebrates [8,9,10]. The absence of this key VNO function makes the discovery of human pheromones even more challenging.

The *Caenorhabditis elegans* dauer pheromone, which is part of an important chemical language throughout this nematode’s lifespan, has long been known. In 1975, Cassada and Russell first reported the existence of dauer larvae, an alternative developmental stage that prolongs survival under environmental conditions that do not support growth [11]. The observation of dauer larvae might have provoked the search for dauer pheromones. In 1982, the first biological evidence of a nematode pheromone was reported by the Riddle group, who showed that a partially purified *C. elegans* extract could trigger dauer formation in L1/L2 larvae [12]. Indeed, this pioneering work inspired worm biologists to continue to search for pure dauer pheromones.

## 2. Structural Diversity of Ascaroside (ascr) Pheromones

### 2.1. Daumone, the First Chemically Characterized Ascr Pheromone

**Figure 1:**
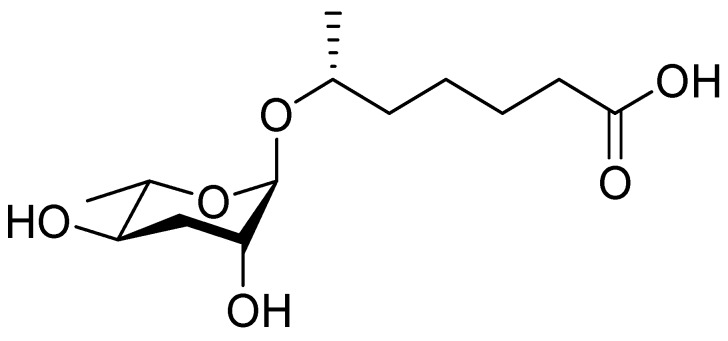
The chemical structure of daumone, the first characterized ascaroside (ascr) pheromone (**ascr#1**), contains an ascarylose sugar and a methylated short-chain fatty acid (mSCFA) linked by an ether bond [(2)-(6R)-(3,5-dihydroxy-6-methyltetrahydropyran-2-yloxy) heptanoic acid] [13].

In 2005, the Paik group isolated and chemically characterized the first *C. elegans* pheromone, which they named dauer pheromone, or daumone (now often referred to as **ascr#1**) [13]. Via an activity-guided purification procedure using 300 L of cultured worms, they isolated pure daumone, which has the molecular formula C_13_H_24_O_6_ and an *M*_r_ of 276 (Figure 1). Determination of the stereochemical structure of purified daumone, [(2)-(6R)-(3,5-dihydroxy-6-methyltetrahydro-pyran-2-yloxy) heptanoic acid], revealed that it contains one ascarylose (a 3,6-dideoxy sugar also known as rhamnose) linked to the C7 of a methylated short-chain fatty acid (mSCFA) (Figure 1).

They also demonstrated that natural and chemically synthesized daumone could equally induce dauer formation in the wild-type *C. elegans* laboratory strain (N2) and in *Caenorhabditis briggsae*. The discovery of daumone, which is indeed a bona fide signaling molecule, not only settled a long-time dispute as to whether the *C. elegans* pheromone acted as a signal or a crowd cue [14], it also opened a new avenue for investigating the chemical biology of ascr pheromones on molecular and system-wide scales. As additional dauer pheromone derivatives (collectively called ascarosides) were identified, daumone was later renamed ascaroside #1 (**ascr#1**) as per Edison’s suggestion [15], which was based on the presence of an ascarylose sugar moiety linked to an mSCFA. In this review, we use “ascr pheromones” rather than ascarosides to distinguish between the pheromones and non-pheromonal ascr derivatives or metabolites, consistent with the terminology used for steroid hormones (i.e., steroids vs steroid hormones). This distinction is important, given that more than 200 ascaroside-like compounds with unknown functions have now been identified via metabolomic methods [16,17].

Historically, non-pheromonal ascarosides were first identified among the neutral lipids of parasitic nematodes such as *Ascaris lumbricoides* and *Parascaris equorum* [18,19]. These compounds typically consist of a glycone moiety (one or two ascarylose units, i.e., a 3,6-dideoxy sugar) and an aglycone moiety, a very long chain fatty acid (VLCFA) that contains greater than or equal to 25 carbon atoms [20]. They were mainly recovered from the eggs and reproductive tract tissue of female *A. lumbricoides* nematodes, and they were shown to confer the eggs with chemical resistance against external toxic insults [21]. Therefore, unless otherwise stated, our discussion will be limited to ascr pheromones and their potential neuronal functions.

### 2.2. Identification of Diverse Ascr Pheromones in Nematodes

**Figure 2:**
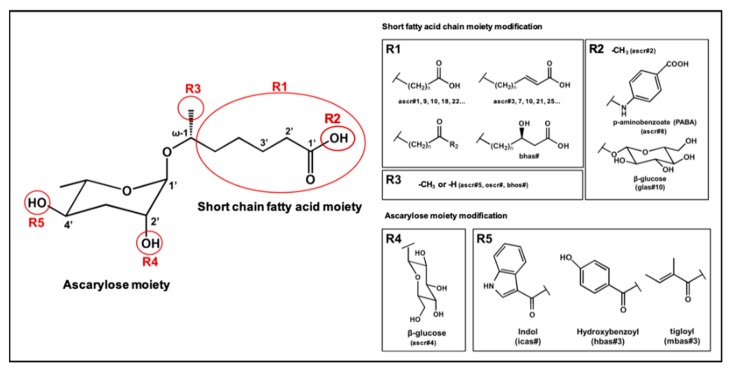
Structural diversity among the known ascr pheromones. Several ascr pheromone analogs modified at various positions (red circles labeled R1–5) have been identified [13,16,17,22,23,24,25,26,27].

In the 15 years since the discovery of the first ascr pheromone, several groups have intensely investigated their chemical biology. For instance, the Clardy group energized the pheromone research community by identifying two additional ascr pheromones (i.e., **ascr#2** and **3**) in cultured worms [22]. **Ascr#2 and 3** contain essentially the same structural backbone as **ascr#1** (**C7**-SCFA) but differ in the number of carbons in the mSCFA moiety linked to the 3,6-dideoxy ascarylose sugar (**ascr#2**: **C6-**mSCFA with a methyl ketone, **ascr#3**: α-β unsaturated **C9**-mSCFA) (Figure 2). To distinguish the ascr pheromone families in this article, we classify them into two groups: the simple ascr pheromones, which contain only ascarylose and mSCFA, and the modular ascr pheromones, which contain modified ascarylose. Other simple and modular ascr pheromones have now been identified and characterized [16,23,24,25,26,27]. In particular, the Schroeder group detected small amounts of several ascarosides (i.e., **ascr#6.1**, **6.2**, **7**, and **8**) among the metabolites of the wild-type N2 strain by comparing its two-dimensional nuclear magnetic resonance spectrum with that of the ascaroside biosynthesis-defective *daf-22(m130)* strain [25]. These ascr pheromones contain an unsaturated seven-carbon mSCFA linked to a *p*-aminobenzoate subunit (i.e., **ascr#8)** or β-glucose (i.e., **glas#10**) [25] (Figure 2).

The indole carboxy (IC) ascarosides (icas, e.g., **icas#1**, **3**, **7**, **9**, and **10**) contain a unique indole-3-carbonyl unit attached to the 4′-position of ascarylose [24,27] (Figure 2). Profiling of worm extracts via MS/MS fragmentation and GC-EIMS led to the identification of approximately 200 additional ascr derivatives [16,17]. Notably, structural variations were found in the carbon chain lengths of the mSCFA moiety (e.g., **ascr#18**, **21**, **22**, **and 25**). More examples are the presence of hydroxybenzoyl (**hbas#3**) or 2-methyl-2-butenoyl moieties (**mbas#3**) attached to the 4′-position of the ascarylose, ω-linkages at the terminal carbons of the mSCFA moieties, and 2′-hydroxylation of the mSCFA (Figure 2).

Most functionally characterized ascr pheromones are ω-1 linked, i.e., a methyl group is attached to the C1 position at the link between the mSCFA and the ascarylose; however, ω-linked ascr pheromones lacking this linkage have also been reported [16,23] (Figure 2). The structural diversity in the mSCFA is likely generated via the multi-cycled peroxisomal β-oxidation that occurs during ascaroside biosynthesis, although other mechanisms are possible [16,28,29,30,31,32,33,34,35,36]. Some structural derivatives of ascr pheromones contain other functional groups (e.g., a methyl group, amino acid precursor, glucose, or benzoyl group) linked to the 2′- or 4′-position of the ascarylose moiety or to the 1′-position of the mSCFA moiety, generating a collection of highly diverse ascaroside structures (Figure 2). It is worth noting that ascr pheromone-like derivatives have also been identified in other nematode genera [37,38,39,40,41,42]. Moreover, the Sternberg group showed that the difference of ascaroside blends between many nematode species was observed with respect to variance of ascr pheromone composition [39]. As most known ascr derivatives share a common structural backbone but differ in their mSCFA moieties or ascarylose modifications (Figure 2), determining their individual functions will be a daunting task.

## 3. Ascr Pheromone Biosynthesis and Metabolic Regulation

### 3.1. Ascr Pheromone Biosynthesis

Initially, it was proposed that the ascr pheromone precursors are produced via two distinct reaction pathways, peroxisomal β-oxidation for the SCFA moiety and de novo biosynthesis for the ascarylose moiety (both the simple and modified forms). To produce mature, active ascr pheromone, the SCFA and ascarylose moieties would then be conjugated by UDP (uridine diphosphate)-glucuronosyl transferase (UGT) [29]; however, an alternative pathway has now been proposed. In this alternative pathway, a VLCFA-conjugated ascarylose is first produced and then subsequently subjected to peroxisomal β-oxidation to produce active ascr pheromone [43,44]. This proposal was supported by genetic screens and metabolomic experiments. In *maoc-1*, *dhs-28*, and *daf-22* mutant strains, most of the ascarosides with fatty acid chain lengths of less than nine carbons are not synthesized, whereas non-pheromonal FA-conjugated ascarylose (e.g., VLCFA-, VLCFA-CoA-, and LCFA (long-chain fatty acid linked ascarylose)) accumulates in the worm body [16,17,29,30,43].

Naturally, the source of the ascarylose moiety was an interesting question. The Paik group previously demonstrated that the ascarylose was not derived from the *Escherichia coli* consumed by the worms, but rather that it was de novo synthesized [29]. Sorting out this issue was necessary because ascarylose (a glycoconjugate of ascaroside) is found in the lipopolysaccharide (LPS) of Gram-negative bacteria, and it represents a unique class of sugars with a 3,6-dideoxy sugar structure [45]. In bacteria such as *Yersinia pseudotuberculosis*, ascarylose is produced via a continuous chain of five enzymatic reactions in which CDP-D-glucose is produced from glucose-1-phosphate [46,47,48]. However, in the course of studying egg shell formation, a gene responsible for ascarylose biosynthesis was found in *C. elegans* [49], supporting the earlier argument in favor of de novo ascarylose biosynthesis [29]. However, additional work is needed to elucidate the detailed mechanism of this step in *C. elegans*.

UGT might be an ideal candidate for catalyzing the conjugation of ascarylose to VLCFAs, as occurs during detoxification reactions in mammals [50,51]. The basis of this prediction is that during detoxification, UGT transfers the monosaccharide glucuronic acid to lipophilic metabolites (e.g., steroids and bile acids) and xenobiotics (e.g., environmental toxins) to render them water-soluble for release [52,53,54]. However, it remains unknown in *C. elegans* whether an enzyme similar to UGT might catalyze the linkage of fatty acids to ascarylose or cooperate with other enzymes to specifically synthesize ascarosides. For instance, the enzyme encoded by *dgtr-1*, which is involved in egg shell formation, is also thought to be required for ascaroside synthesis because of its homology to the DGAT2 family of acyl-CoA:diacylglycerol acyltransferases, which catalyze the addition of fatty acyl-CoA to diacylglycerol to form triacylglycerol [49].

**Figure 3:**
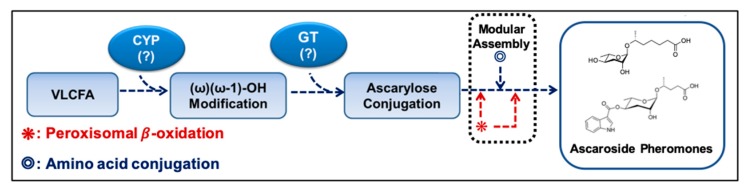
Schematic working model of the ascr pheromone biosynthetic pathway. CYP alters the very long chain fatty acid (VLCFA) produced via elongation of C16 or C18 fatty acids to produce ω-1 or ω-oxygenated VLCFA substrates. Ascarylose is then linked to the ω-1- or ω-oxygenated VLCFAs to form VLCFA-linked ascarosides. Finally, an ascr pheromone containing a shortened fatty acid chain is produced via peroxisomal β-oxidation. In this case, amino acid precursors are linked to specific ascr pheromones. CYP: cytochrome P450, GT: glucuronyltransferase. (?): Names of these enzymes are not known in *C. elegans*.

During the biosynthesis of modular ascarosides (e.g., **icas**, **mbas**, **hbas**, and **osas**), several organic moieties (e.g., amino acid metabolites) are attached to the 4′-position of ascarylose. Using deuterium-labeled tryptophan and axenic in vitro culturing, the Schroeder group found that the indole carbon atom of **icas** is derived from l-tryptophan, while the 4-hydroxybenzoyl group of **hbas** is derived from l-tyrosine or l-phenylalanine. Furthermore, the tigloyl group of **mbas** and the octopamine succinyl group of **osas** are derived from l-isoleucine and l-tyrosine, respectively [16,55]. It has also been suggested that lysosomal ACS-7, an acyl-CoA synthase, catalyzes the linkage of indole-3-carboxy (**icas**) or N-succinyl octopamine groups to ascr [32]. However, the Butcher group showed that ACS-7 appears to transport **icas** to the peroxisomes during the biosynthesis of the short-chain ascaroside **icas** [36]. This dispute on the function and cellular location of ACS-7 remains to be resolved. Based on the findings discussed above, a working model for the biosynthesis of both simple ascr (no attached organic moieties) and modular ascr (various attached organic moieties) in *C. elegans* can be proposed (Figure 3). In this scheme, cytochrome P450 generates (ω-1) or ω-oxygenated VLCFA or LCFA precursors that are then linked to ascarylose to form FA-linked ascarosides (e.g., LCFA). The FA-linked ascarosides then enter the peroxisomal β-oxidation pathway to produce active mSCFA ascr pheromones [16,49].

Peroxisomal β-oxidation is a central metabolic pathway in animals that supplies SCFA components for energy production in mitochondria as well as the main carbon chain precursors for ascr pheromones. The presence of peroxisomes in the intestine and hypodermis of *C. elegans* and the target signals of their peroxisomal proteins have been revealed [56,57]. This topic has been covered in detail by recent publications, and the field is still evolving; therefore, this discussion focuses on important developments related to the production of the mSCFA moieties used in ascr pheromones, as the mSCFAs are a key driver of the structural and functional diversity of ascr pheromones. Research on the ascr biosynthetic pathway has progressed well since the discovery of the nematode acyl-CoA oxidases (ACOX-1 or ACOX-1.1) [30]. ACOXs catalyze the first reaction of peroxisomal β-oxidation by producing enoyl-CoA from acyl-CoA, and they contribute to maintaining the ascr pheromone pool synthesized in response to sudden environmental shifts [30]. Some ascr pheromones (i.e., **ascr#2**, and **3**) are not synthesized by the *acox-1* (*ok2257*) mutant strain [30], whereas the synthesis of others (**ascr#1**, **9**, **10**, **oscr#9**, and **10**) is elevated [16]. These observations suggest that the *acox-1* gene produces multiple ACOX isoforms, which were later found to have different substrate specificities [33,34,35]. The Butcher group used CRISPR/Cas9 genome editing to elegantly produce various mutant derivatives of the ACOX isoforms and found that the different ACOX isoforms can form various homo- and heterodimers with distinct substrate preferences that produce different ascr pheromones. For example, the ACOX-1.1/ACOX-1.4 heterodimer produces **ascr#1** while the ACOX-1.1/ACOX-1.3 heterodimer produces **ascr#2** [35]. This mechanism for the biosynthesis of such diverse ascr pheromones by the ACOX isoforms is supported by the observation that ACOXs might act on (ω-1)- and ω-oxygenated VLCAs prior to their cyclic stepwise breakdown during peroxisomal β-oxidation. Furthermore, this finding also confirms an earlier report that the ACOXs help to define the ascr pheromone population produced by *C. elegans* [30]. For the second and third reactions of the peroxisomal β-oxidation pathway, MAOC-1 hydrates enoyl-CoA to produce hydroxyacyl-CoA and DHS-28 dehydrogenates hydroxyacyl-CoA to produce 3-ketoacyl-CoA [16,28,29]. Finally, mature mSCFA-containing ascr pheromones are produced via the thiolase activity of DAF-22, a homolog of human SCPx.

### 3.2. Transcriptional Regulation of Ascr Pheromone Biosynthesis by Environmental Stressors

Although sequence of the biosynthesis of ascr pheromones is known well, it remains unknown how these enzymes are transcriptionally regulated by environmental changes (e.g., temperature increases, nutrition deprivation). To address this question, it was essential to quantify the levels of the approximately ~200 ascr derivatives currently known in *C. elegans*, and to accurately measure the changes in the levels of the ascr pheromones under various physiological states via a standard quantification method [58,59,60]. The Paik group developed the “PheroQu” method, a multiple reaction monitoring (MRM)-based ascr pheromone quantification method that uses ultra-performance liquid chromatography coupled to mass spectrometry (MS) with only 20 worms. This method enables accurate quantification of the levels of various ascrs in the worm body and in the medium during larval development [59]. With this method, it was found that the biosynthesis of several ascr pheromones (**ascr#1-3**) is robustly influenced by developmental stage, growth condition, and environmental stress (e.g., heat) throughout the life cycle [59].

Upon an increase in ambient temperature, the levels of ascr pheromones increase up to two-fold [30]. It was later found that heat-shock factor 1 (HSF-1) regulates the transcription of ascaroside synthesis genes (e.g., *acox-1*, *dhs-28*, and *daf-22*) in response to external temperature. This finding was supported by chromatin immunoprecipitation assays and increased production of chemically detectable ascarosides (e.g., **ascr#1** and **3**) [31]. Based on this observation, it appears that *C. elegans* requires transcriptional regulation to ensure that a sufficient ascr supply is available upon encountering sudden environmental changes or stress signals, such as poor nutrition or high population density, to prepare for dauer entry. Related to this concept, the Butcher group recently reported that poor nutrition and high temperature can lead to the transformation of one type of ascr (e.g., aggregation-inducing medium-chain **icas**) into another type (e.g., dauer-inducing short-chain **icas**), providing evidence of flexibility in the structure and function of ascr pheromones in response to environmental stress [33,36]. Thus, via combinatorial usage of the products of the *acox* gene family, *C. elegans* has multiple options for adapting to new environments without expending metabolic energy and resources [36].

## 4. Pleiotropic Neuronal Functions of Ascr Pheromones

### 4.1. Roles of Ascr Pheromones in Development and Aging

The ascr pheromones influence a variety of functions in the chemosensory neurons that control development, aging, and behaviors in conspecific individuals. Depending on their concentration in the media, they also trigger other important behaviors (e.g., dauer-induction, lifespan extension, mating attraction, repulsion, aggregation, and foraging) that are essential for survival under stressful conditions [26,36,61,62]. Perhaps the best-known function of ascr pheromones is their ability to induce dauer entry, which is a unique system for prolonged survival in *C. elegans*. Reports from several groups showed that there are robust changes in the expression levels of various genes in dauer larvae and dauer entry and exit [63,64,65,66]. These findings indicate that ascr pheromones exert their biological functions via some less-characterized signaling pathways involved in neuronal transmission [13,15,26].

By taking advantage of the availability of ascr pheromones, the Paik group characterized the real-time metabolic molecular landscape during dauer formation. These data revealed the metabolic changes underlying the worm’s adaptation during the developmental shift to diapause. They measured the genome-wide gene expression changes via DNA microarrays that cover 22,250 unique genes. Their results suggested the presence of a unique adaptive metabolic control mechanism that requires both stage-specific expression of specific genes as well as tight regulation of different modes of fuel metabolite utilization to sustain the energy balance for prolonged survival under adverse conditions [63]. A comprehensive web-based dauer metabolic database for *C. elegans* is available (www.DauerDB.org) for use by the research community and might be broadly useful as a molecular atlas for related nematodes. In addition, using the chemically available pure ascr pheromones, the Lee group routinely produced *C. elegans* dauer larvae and explored that IL2 neurons mediate a phoretic behavior of dauer larvae, called nictation [67]. Furthermore, the same group also characterized nictation as a means of dispersal and survival strategy under harsh conditions through interspecific interaction of *C. elegans* dauer larvae [68].

The clarification of the molecular pathways involved in dauer induction raised questions about the presence of ascr pheromone receptors, which should mediate pheromone sensing to elicit dauer entry. At least three putative pheromone receptors that directly trigger the relevant signaling pathways have been identified in several nematode species. The first ascr pheromone receptor was reported by the Sengupta group, who discovered that the G protein-coupled receptors (GPCRs) SRBC-64 and SRBC-66 are expressed in ASK neurons where they are required for pheromone-induced dauer formation [69]. However, *srbc-64(tm1946)* and *srbc-66(tm2943)* mutant worms failed to form dauer larvae in response to **ascr#1–3** but entered the dauer stage normally in response to **ascr#5** [69]. The decrease in the calcium level in the ASK neurons in response to ascr pheromone observed in adult wild-type worms was not detected in the *srbc-64(tm1946)* and *srbc-66(tm2943)* strains. These mutants did not exhibit long-term responses to pheromones, indicating that other pheromone receptors function via competing signaling cascades depending on the developmental stage [70]. The Bargmann group reported that two other GPCRs, SRG-36 and SRG-37 (which belong to the serpentine receptor class), might act as **ascr#5**-specific ascr pheromone receptors that relay the same dauer entry signals in the ASI neurons [71]. They took advantage of two *C. elegans* strains (LSJ2 and CC1) that had been propagated for long periods of time in liquid axenic media that, unlike the wild-type N2 strain, did not form dauer larvae in response to ascr pheromones (**ascr#1**, **2**, **3**, and **5**). Quantitative trait locus (QTL) mapping and whole-genome sequencing revealed single-nucleotide polymorphisms in *srg-36* and *srg-37* in LSJ2 and CC1, respectively, that specifically prevented the response to **ascr#5**. In *C. briggsae*, another nematode species, the receptor encoded by an *srg* gene paralogous to *srg-36* and *srg-37* responds to **ascr#5** [71]. These results indicate that remodeling of the chemoreceptor repertoire in nematodes allows adaptation to the external environment and that changes in paralogous genes may have common effects across species. In 2012, the Riddle group found that DAF-37 and DAF-38 (also GPCRs) function as a heterodimer to respond to ascr pheromones [72]. DAF-37 responds specifically to **ascr#2**, and its expression in ASI neurons regulates **ascr#2**-mediated dauer formation, whereas its expression in ASK neurons regulates adult behavior. DAF-38, on the other hand, plays a cooperative role in sensing **ascr#2**, **3** and **5** [72]. Other candidate molecules involved in pheromone-induced dauer formation were identified using a forward genetic screen; however, they seem to function in pheromone signaling rather than as pheromone receptors [73,74]. The findings that different pheromone-responsive receptors are expressed in different neurons suggest that additional receptor molecules in other neurons might remain to be identified.

The Scheroder group recently found that **ascr#2**, a ligand of the DAF-37 ascr receptor, mediates an approximately 20% lifespan extension in a sirtuin-dependent manner [75]. This finding revolutionized our thinking on how dauer formation is involved in lifespan extension in *C. elegans*. This new concept, known as ascr-mediated increases of lifespan (AMILS), represents a new paradigm for chemosensation-based non-dauer lifespan extension as it is independent of DAF-16-governed insulin signaling and DAF-12. Given the availability of other ascr pheromones, it would be interesting to investigate whether AMILS is specific to **ascr#2** or whether it exists in other nematode genera or can be regulated by other ascr pheromones.

### 4.2. Neuronal Effects of Ascr Pheromones on Nematode Social Behaviors

**Figure 4:**
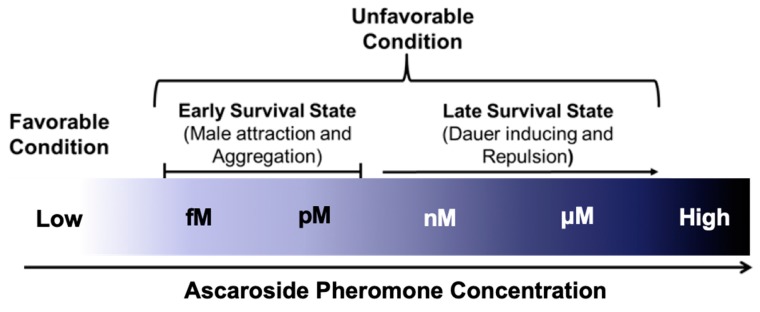
The pleiotropic neuronal functions of major ascr pheromones (e.g., **ascr#1**–**3**) exerted at their environmental concentrations.

As described above, ascr pheromones have a wide spectrum neuronal functions that not only mediate dauer entry, but also influence adult behaviors and phenotypes, including lifespan extension. For example, very low concentrations (fM–pM) of ascr pheromones attract males, whereas higher concentrations (nM–μM) promote dauer entry [26] (Figure 4). **Ascr#3**, in particular, seems to act as a strong male-attracting pheromone, and various concentrations of **ascr#2**–**4** appear to exhibit strong synergistic roles in amphid single-ciliated sensory neurons (ADF/ASK) and cephalic companion neurons (CEM) [26,76,77]. **Ascr#8** is also an important male-attracting pheromone at both low and high concentrations [25,76]. These findings clearly confirm that ascr pheromones have neuronal functions that trigger diverse behaviors to ensure prolonged survival in response to environmental changes.

Interestingly, although **ascr#1**–**3** induce dauer entry of L1 worms at higher concentrations (nM–μM), similar concentrations act as chemorepellents after the L1 stage that stimulate hermaphrodite repulsion [26,62,77,78,79,80]. These observations suggest that these pheromones act in a concentration-dependent and stage-specific manner. These repulsive responses appear to be transmitted via the GPA-3-DAF-16/FOXO signaling pathway in sensory neurons, and they affect long-term memory via glutamate signaling regulated by DAF-16 [78]. Note that this behavior is distinct from male attraction behavior because the genetic sex modulates the sensitivity of the ADF neurons to ascr pheromones [77]. The **ascr#3**-dependent avoidance behavior is stimulated by **ascr#3** sensing in the ADL neurons followed by signal propagation to the interneurons, which then regulate the magnitude of the behavioral changes stimulated by pheromone contact in relation to feeding state or early larval development [79,80]. Furthermore, **mbas#3** (an ascaroside linked to a tigloyl group) and **osas#9** (an ascaroside linked to a succinyl octopamine group) also have repulsive effects similar to those of **ascr#3** and **icas#3** [55,81].

At low concentrations (< 10 nM), **ascr#2**, **3**, and **5** can attract hermaphrodites only in specific social strains or strains lacking NPR-1 (e.g., the *npr-1(ad609)* mutant), an important regulator of aggregation behavior [62]. At low concentrations, some IC group-containing ascr pheromones (e.g., **icas#1**, **icas#3**, and **icas#9**) induce aggregation in solitary N2 hermaphrodites as well as in naturally isolated social strains (e.g., CB4856 and RC301), while they induce male attraction at higher concentrations [27]. These responses require the ASK sensory neurons and downstream AIA neurons, but not the RMG neuron required for attraction in *npr-1(ad609*) mutants as previously reported. Like the icas pheromones, **ascr#1**, **2**, **3**, and **5** can act as chemorepellents or aggregation-inducing pheromones, suggesting that their activity is determined by their environmental concentrations. At low concentrations, they induce attraction, whereas at higher concentrations they induce repulsion. One group reported that this behavioral change also depends on the oxygen concentration [82]. In this study, the authors found that RMG neurons control the oxygen concentration via the URX neurons, resulting in switching between attraction signals in ASK neurons and repulsion signals in ADL neurons. The discovery of the **icas#9** receptors, encoded by *srx-43* and *srx-44*, via QTL mapping and whole-genome sequencing [61,83] revealed that SRX-43 is expressed in ASI neurons, whereas SRX-44 is expressed in ASJ and ADL neurons, and that roaming behavior is determined by the site of their expression [83].

In several asexual species, the rate of sexual reproduction increases in stressful environments, functioning as a survival strategy to generate genetic variation via recombination during outcrossing [84,85,86,87,88,89,90,91,92]. In *C. elegans*, ascr pheromones induce male mating or aggregation behavior in the early survival state. For example, two naturally occurring strains (CB4856 and JU440) exhibit increased male frequency during the dauer stage that is not observed in the N2 laboratory strain N2. This effect is due to an increase in the male mating rate and increased male survival during the dauer period [93]. The male attraction behavior in response to ascr pheromones is thought to induce an increase in male frequency in dauer-inducing environments [15]; thus, it is likely that larger male populations are beneficial for survival in unfavorable external environments. One study reported that the hermaphrodite reproductive rates of some other naturally isolated strains are regulated by secreted pheromones [94]. In fact, **ascr#3** and **10** are secreted at different rates by males and hermaphrodites [95]. A combination of ascr pheromones secreted by males has been reported to not only affect the hermaphrodite reproductive system, but also to increase heat stress resistance [96]. This male-secreted pheromone also has a male-killing effect, thereby regulating the population size of the species [97]. In sum, the functions and structure of some ascr pheormones are listed in Table 1.

The concentrations of the ascr pheromones produced by worms and their main functional changes in response to external environmental conditions are outlined in Figure 4. Under favorable conditions, the ascr pheromone concentrations are too low to exert any effects, perhaps due to other environmental factors. However, ascr pheromone synthesis gradually increases as worms encounter unfavorable stress conditions (e.g., high temperature, food limitation, and high population density) [30,59]. It has been hypothesized that ascr pheromones stimulate male mating or aggregation at relatively low concentrations under normal growth conditions, while under stressful conditions that trigger increased ascr pheromone production (and thus higher concentrations), worms may exhibit a repulsive response to ascr pheromone and enter the dauer state. However, the structural basis for the functional differences between ascr pheromones has not yet been clarified.

## 5. Implications of Ascr Pheromone Metabolism in Neuroprotection

### 5.1. Implications of Ascr Pheromone Biosynthesis Gene Deficiencies in Neuronal Disorders

Several ascr pheromone biosynthesis defects have been identified in mutant worms deficient for peroxisomal β-oxidation enzymes [16,28,29,30,31,32,33,34,35,36,98]. The physiological consequences of impaired DAF-22-dependent peroxisomal *β*-oxidation of VLCFAs or fatty acyl-CoAs involved in the production of various aglycone units (mSCFAs with less than nine carbon atoms) required for pheromone biosynthesis indicate that peroxisomal *β*-oxidation of VLCFAs is an essential detoxification process for clearing harmful peroxisomal fatty acids to maintain cellular homoeostasis. This function indicates that ascr pheromones not only regulate stress avoidance, they also maintain cellular homeostasis via the production of excretable FA-ascarylose conjugates (ascarosides) [29]. Here we examine the pleiotropic neuronal functions of ascr pheromones from two different angles, ascr metabolic deficiency and chemotactic responses.

**Figure 5:**
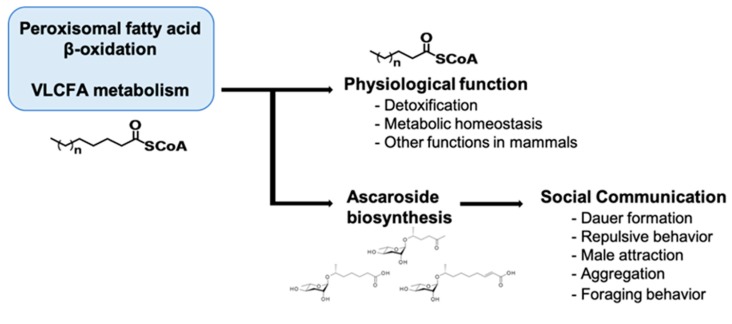
A schematic diagram for the dual role of peroxisomal fatty acid (FA) β-oxidation. By shortening VLCFAs, peroxisomal fatty acid β-oxidation can exert physiological functions such as detoxification and maintenance of metabolic homeostasis. In *Caenorhabditis elegans*, shortened FAs are used to synthesize ascr pheromones, which are important for social communication.

In mammals, it is well known that peroxisomal malfunctions induce developmental defects and neurodevelopmental diseases. These diseases include Zellweger syndrome (ZS) and X-linked adrenoleukodystrophy (X-ALD), which involve severe neurological problems that often lead to death in infants and young children [99,100,101,102,103,104]. In humans, a single defect in an enzyme involved in peroxisomal fatty acid β-oxidation leads to ZS, which involves abnormal symptoms such as neonatal hypotonia, craniofacial dysmorphia, seizures, and developmental delay [100,103,104,105]. Mechanistically, it was suggested that the defect in peroxisomal fatty acid β-oxidation results in the accumulation of VLCFAs in the form of triacylglycerols, which are harmful to animals [29,105]. Furthermore, decreased docosahexaenoic acid (DHA; C22:6 (n-3)) levels, plasmalogen depletion, and abnormal neurons myelination (e.g., degenerative loss of myelin (demyelination) or abnormally formed myelin (dysmyelination)) have been suggested to underlie the neuropathologies associated with peroxisomal disorders [106]. In *C. elegans*, ascaroside biosynthesis appears encompass two important physiological roles that affect the worm’s quality of life: (1) a social function in which pheromone production affects the behavior and physiology of other individuals, and (2) protection of metabolic homeostasis via the removal of toxic VLCFAs in peroxisomes (Figure 5) [29].

In addition to neurodevelopmental defects, deficiencies in peroxisomal fatty acid β-oxidation seem to be related to other pathologies. In *C. elegans*, animals deficient in peroxisomal fatty acid β-oxidation, such as the *dhs-28(tm2581)* and *daf-22(ok693)* mutant strains, exhibit short lifespans and developmental delays, and are more susceptible to environmental stresses, limiting the worm’s survival under harsh conditions [29,107]. In particular, it has recently been suggested that peroxisomal fatty acid β-oxidation has distinct functions in neuronal cells for maintaining normal development and nervous system function [101,106,107]. More interestingly, it was revealed that neuronal peroxisomal fatty acid β-oxidation has an important cell-autonomous function to regulate neuroendocrine signaling activities [107]. The *C. elegans* SCPx gene *daf-22* is expressed in a subset of chemosensory neurons, i.e., the ASK neurons, where its activity is required for exogenous pheromone-induced dauer entry [107]. A deficiency in neuronal peroxisomal fatty acid β-oxidation activates the lipid-induced endoplasmic reticulum (ER) stress response, which then increases the expression of insulin-like peptides in neurons and abnormally enhances insulin/IGF-1 signaling activity to eventually interrupt dauer entry [107]. Meanwhile, ER stress-mediated dauer diapause is also regulated by other sensory neurons, such as the ASI neurons [108]. It has been suggested that the mutated DAF-28 peptide in the *daf-28*(*sa191*) mutant strain triggers ER stress and activation of the unfolded protein response (UPR) to induce constitutive dauer entry [108,109,110,111].

**Figure 6:**
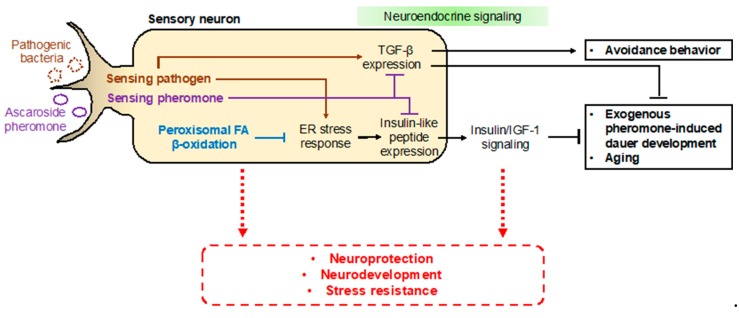
The protective ways in the sensory neurons and their outputs. In *C. elegans*, ascr pheromone sensing affects the expression of neuropeptides, such as insulin and TGF-β. Peroxisomal fatty acid β-oxidation in sensory neurons regulates neuroendocrine signaling (e.g., insulin/IGF-1 signaling) via regulation of insulin-like peptide expression by suppressing the lipid-induced endoplasmic reticulum (ER) stress response. By regulating insulin/IGF-1 signaling, peroxisomal fatty β-oxidation controls both exogenous pheromone-induced dauer entry and aging. Furthermore, pathogens regulate TGF-β expression and ER stress via unfolded protein responses (UPRs). TGF-β expression triggered by pathogens stimulates avoidance behavior. Similarly, such signaling in the nervous system can influence neuroprotection, neurodevelopment, and stress resistance, either directly or via neuroendocrine signaling pathways.

From these studies, it can be inferred that peroxisomal fatty acid β-oxidation is important for neuroprotection via the regulation of metabolic homeostasis (e.g., balance in fatty acid levels), myelination of neuronal cells, and the regulation of cellular signaling; these neuroprotective functions could influence aging, neurodevelopment, and stress resistance. Therefore, it is important to investigate the mechanisms underlying the roles of neuronal peroxisomal fatty acid β-oxidation in neuroprotection and aging in the future. It would also be worthwhile to elucidate the links between neuronal peroxisomal disorders and alterations in neuronal function and neurodevelopment (Figure 6).

### 5.2. Implications of Ascr Pheromone Signaling in Chemotactic Responses

Ascr pheromones induce a variety of behaviors [112]; however, these behaviors are controlled not only by the ascr pheromones but also by various other associated factors and environmental conditions. In general, food signals play important roles in determining behaviors and developmental choices in the presence of ascr pheromones in *C. elegans*. For example, calcium/calmodulin-dependent protein kinase I (CMK-1) regulates pheromone-mediated dauer entry in ASI/AWC neurons depending on the feeding state, although not directly via a pheromone-binding receptor [113]. Furthermore, gut-to-neuron signaling induced by feeding conditions affects TGF-β and insulin expression via target of papamycin complex 2 (TORC2), which leads to dauer entry or behavioral changes [114]. Repulsive behavior in response to feeding status is also induced by pheromone-mediated insulin signaling [80]. The combination of these two signals determines the choice between dauer entry or progression to the reproductive state via downstream regulation of DAF-12 and the associated *let-7* microRNA family and hunchback-like-1 (HBL-1) [115]. Ascr pheromones are also involved in chemotactic behavior by regulating endogenous peptide signaling [116]. *C. elegans* exhibits chemotactic attraction toward odorants such as benzaldehyde; however, after prolonged exposure, the chemotactic behavior shifts to a dispersion behavior, and this shift is called olfactory adaptation or food-odor associative learning. The results of the study of Yamada et al. also suggest that NEP-2 (a homolog of the extracellular peptidase neprilysin) and SNET-1 (an NEP-2 suppressor peptide) regulate olfactory adaptation, and that an ascr pheromone that inhibits *snet-1* expression is essential for olfactory adaptation [116].

Factors associated with ascr pheromones and their sensing have also been implicated in other physiological processes, such as aging [117,118,119,120]. This change in longevity is not only affected by ascr pheromones, but rather it is also influenced by a combination of other factors, including nutritional state and population density [75,121]. These pheromones act as a kind of warning signal by which *C. elegans* is informed in advance of ongoing changes in growth conditions (e.g., the ratio between food and pheromones). Triggering of this warning signal is also caused by other factors in addition to ascr pheromones. Typically, pathogen-induced avoidance in *C. elegans* has been studied in the context of the innate immune system [122,123]. Interestingly, it appears that the signaling in response to exposure to food bacteria and pathogenic bacteria and the downstream effects are similar, with the difference being the toxicity of the organisms to the worms [124]. Several factors simultaneously play important roles in ascr pheromone-mediated signaling and pathogen avoidance. First, NPR-1, which controls aggregation via ascr pheromones [62], also plays an important role in pathogen avoidance [125,126,127]. Like pheromones, pathogens are also recognized by sensory neurons [128,129]. Furthermore, the TGF-β ligand and insulin, which also play important roles in dauer entry, also appear to be involved in pathogen avoidance [130,131,132]. However, DAF-7, a TGF-β ligand, acts in the ASI/ASJ neurons during pathogenic avoidance but primarily in the ASI neurons during ascr pheromone sensing [130,131]. Finally, ER stress or UPR activation in sensory neurons can also be induced by pathogens [133,134,135,136]. It is plausible to predict that these physiological effects might involve the same factors to promote the survival of the nematode (Figure 6). Indeed, it has been reported that the use of ascr pheromone in a mammalian system has a therapeutic effect on hepatic inflammation [137,138]. Furthermore, ARTD, a combination of artemisinin and ascr pheromone, can also be used as an effective therapeutic agent in osteoclasts, where it shows a potent cancer inhibitory effect [139]. Thus, this relationship deserves further investigation in the future.

## 6. Conclusions and Future Directions

In this comprehensive review, we have highlighted some of the major achievements from the past 15 years since the discovery of the first ascr pheromone (**ascr#1**) [13]. The rapid developments in the ascr field have increased the depth of our knowledge with respect to biosynthetic pathways, ascr receptor-mediated neuronal signaling pathways, and potential neuro-physiological effects in animals. We would also like to add a few words on our views of the future of the ascr pheromone field.

(i) Translational research: Given that their biosynthesis has been thoroughly investigated, now is a good time to construct a chemical biology map or database to catalog the structure-function relationships of the more than 200 members of the ascr family. Since some factors involved in ascr biosynthesis also have important neuronal functions in mammals, translation of what we know about nematode ascr pheromones into studies of metabolic diseases might be a promising future step. Some physiological functions of ascr pheromones are also involved in mammalian aging and disease; thus, these pheromones may have implications in human disease. It will also be interesting to unravel the roles of **ascr#1** in disease model animals or mammalian cells [137,138,139].

(ii) Neuronal pheromone sensing and signaling: Ascr pheromone biosynthesis and their recognition and processing are equally interesting. Previous studies showed that pheromone sensing occurs in sensory neurons, and three receptors specific to some ascr pheromones have been found. However, as the number of newly discovered ascr pheromones increases, how they are sensed and responded to via potential common sensing and signaling pathways remains to be resolved. For example, several GPCRs act as ascr pheromone receptors; however, additional GPCRs have been found in other species [140,141]. Furthermore, several physiological effects induced by ascr pheromones are synergistic, i.e., single pheromones do not always act alone [23,26]. Thus, ascr pheromone sensing and signaling are likely complex and elaborately intertwined and untangling of these knots could provide important clues for understanding neuronal signaling in other species. Given that different ascr pheromones appear to mediate different behaviors across the nematode species depending on environmental conditions, it is reasonable to ask the question, what is the lowest common denominator that underlies the diverse biological functions of ascr pheromones? Fully addressing this question will require additional research on the chemical biology of pheromones in the future.

(iii) Neuronal ascr signaling and behavior: Ascr pheromones were originally found while searching for the factors that influence dauer entry, and they have since been reported to be involved in various behaviors in addition to dauer entry. Interestingly, the effects associated with ascr pheromones are almost exclusively influenced by external environmental cues, many of which involve stress (e.g., poor nutrition, overcrowding, and heat). Therefore, it will be interesting to clarify the biological links between ascr function and stress responses as well as neuroprotection (i.e., the innate immune response, see Section 5.2.).

(iv) Creation of pheromics:In a literature survey of ascr pheromone publications, we noticed many interdisciplinary pheromone research projects and a boom in omics technologies. Examples include, but are not limited to, molecular genetics, chemical biology, metabolomics, proteomics, and genomics. At this juncture, it could be beneficial to create the field of “**pheromics**” (*pheromone omics*) as a new subset of integrated disciplinary research area within chemical ecology with the goal of establishing and supporting a community of researchers involved in the systematic study of the pheromones of living organisms.

## Figures and Tables

**Table 1:** The functions and structure of some ascaroside pheromones *.

Name	Chemical Structure	Discovered Receptors	Functions	References
ascr#1	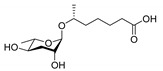	SRBC-64 SRBC-66	Dauer inducing activity Repulsion activity	[13,69,78]
ascr#2	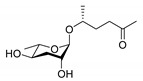	DAF-37 DAF-38 SRBC-64 SRBC-66	Dauer inducing activity Repulsion activity Male attraction activity Foraging activity	[22,26,61,62,69,72,78,82]
ascr#3	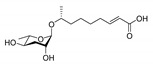	SRBC-64 SRBC-66	Dauer inducing activity Repulsion activity Male attraction activity Foraging activity	[22,26,61,62,69,76,77,78,79,80,82]
ascr#4	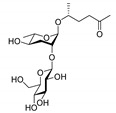	Unknown	Dauer inducing activity Male attraction activity	[26]
ascr#5	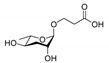	SRG-36 SRG-37	Dauer inducing activity Repulsion activity	[23,62,71,82]
ascr#6.1	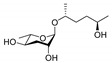	Unknown	Dauer inducing activity	[25]
ascr#8	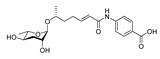	Unknown	Dauer inducing activity Male attraction activity Foraging activity	[25,61,76]
icas#3	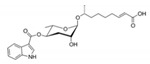	Unknown	Male attraction activity Aggregation activity	[27]
icas#9	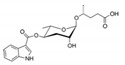	SRX-43SRX-44	Dauer inducing activity Male attraction activity Aggregation activity Foraging activity	[24,27,61,83]
hbas#3	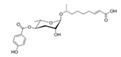	Unknown	Hermaphrodite attraction activity	[16]
mbas#3	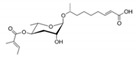	Unknown	Repulsion activity	[16,81]
osas#3	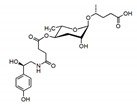	Unknown	Repulsion activity	[55]

* The ascr pheromones listed here were selected based on their identified functions, and citation frequencies.
